# The prevalence and factors associated with malnutrition among infants with cleft palate and/or lip at a hospital in Uganda: a cross-sectional study

**DOI:** 10.1186/s12887-016-0775-7

**Published:** 2017-01-13

**Authors:** Martin Tungotyo, Daniel Atwine, Deborah Nanjebe, Andrew Hodges, Martin Situma

**Affiliations:** 1 Mbarara University of Science and Technology (M.U.S.T), Mbarara, Uganda; 2 Comprehensive Rehabilitation Services for Uganda (CoRSU) Hospital, Kisubi, Uganda

**Keywords:** Cleft palate, Infant, Malnutrition

## Abstract

**Background:**

To determine the prevalence and factors associated with malnutrition among infants with Cleft palate and/or cleft lip (CP+/-L) at Comprehensive Rehabilitation for Uganda Hospital (CoRSU) in Uganda.

**Methods:**

This was a cross-sectional study done on infants with CP+/-L and their caretakers admitted between November 2013 and October 2014 at CoRSU hospital which was the study setting. A questionnaire was answered by the infants’ caretakers. The main outcome measure, malnutrition was defined and classified based on Z-scores obtained using the W.H.O Z-calculator in which weights of the infants in kilograms and lengths in centimeters respectively were placed. The values obtained were expressed as a proportion using all enrolled infants with CP+/-L as denominator. Multivariable analysis was used to determine the risk factors.

**Results:**

A total of 44 infants with CP+/-L were enrolled. Of these, 77% were below 4 months of age and 97.7% were immunized. The male-to-female ratio was 1.06:1. About 59% had unilateral CP+/-L. A total of 93.2% were delivered at term with 69.4% having a birth weight greater than 3 kg. Generally, 68% were malnourished, with the highest burden among females (71.4%), infants below 4 months (73.5%) and those with unilateral CP+/-L (77%). About 57% had moderate-to-severe malnutrition. There was delayed supplementation to breast milk, with cow-milk as the main supplemental feed for all the infants. In the multivariable analysis, factors associated with malnutrition included, having caretakers lacking nutritional information post-delivery (OR = 3.8, p = 0.17), low birth weight (OR = 3.4, p = 0.20), and having less than 10 feeds/day (OR = 4.9, p = 0.09).

**Conclusion:**

CP+/-L infants born in Uganda suffer a high-burden of malnutrition. Preventional strategies are needed with focus on proper feeding information. Research on cost-effective feeds, feeding methods and reasons behind gender disparities in these infants is also required.

## Background

Cleft palate and/or cleft lip (CP+/-L) are among the most common congenital anomalies, with an incidence of 1/1000 live births globally [1] and 0.73/1000 live births in Uganda [2]. Children with CP+/-L are more prone to malnutrition compared to those with only Cleft Lip (CL) [3]. The prevalence of malnutrition among infants with CP+/-L in the literature varies between 30 and 50% [4, 5]. However, information on the prevalence of malnutrition among infants with CP+/-L in Africa is rare.

Malnutrition in these children with CP+/-L is majorly associated with feeding difficulties including the failure to generate sufficient suction pressure during feeding hence affecting the attachment to the breast/artificial nipple, milk extraction, bolus organization and retention of the bolus before swallow initiation [6]. Malnutrition and infection are interlinked as malnutrition is the primary cause of immunodeficiency worldwide and is therefore responsible for the high mortality of children less than five years old who die as a result of infectious diseases [7]. Numerous feeding interventions have been described in the literature for infants born with a cleft palate. A study done in the UK showed a reduction in failure to thrive among infants with CP+/-L after implementation of an early feeding program that involved domiciliary visits, breast feeding support, feeding education and monitoring of growth [6].

Despite the availability of surgical corrective interventions, few CP+/-L children are returned for their cleft palate surgical operation (usually after six months of age) in our setting a phenomenon hypothesized to be due to a likelihood of death before scheduled date of operation potentially due to consequences of malnutrition [8]. Good nutrition is also key in infants with CP+/-L given its role in quickening wound healing post-operatively [9].

However, adequate information on the prevalence and factors associated with malnutrition in CP+/-L infants is still lacking in our setting.

In this study we aimed at establishing the prevalence and associated factors of malnutrition among infants with CP+/-L at CoRSU hospital.

## Methods

### Aim

Determine the prevalence and associated factors of malnutrition among infants with CP+/-L at CoRSU Hospital.

### Study design and setting

Across sectional study was carried out at Comprehensive Rehabilitation Services of Uganda (CoRSU) Hospital, a specialized plastic and reconstructive, as well as orthopedic surgery private facility. It is located along Entebbe road. They offer free cleft surgery and receive patients not only from Uganda but from other countries including South Sudan.

### Study population

A total of 44 admitted infants with CP+/-L between November 2013 and October 2014were consecutively enrolled in the study after obtaining consent from the primary caretakers. There were no primary caretakers that declined to participate or withdraw from the study during the study period.

### Data collection and management

A pre-tested semi-structured questionnaire was developed and administered by trained research nurses to all caretakers of infants with CP+/-L. The questionnaire included caretaker and infant’s baseline characteristics, and infant’s nutritional characteristics. The post delivery nutritional information sought from the caregiver included, whether the caregiver had been advised on any techniques, positions and options available for feeding their infant. Information that was also sought regarding the feeding of the infants included the type of feed and how often it was given. Infant’s clinical examination including type of cleft palate and anthropometry measurements was done by an experienced doctor. Anthropometry included weights and lengths done to the nearest 0.01 kg and 0.1 cm respectively using a digital weighing scale and a length board. Infants who were found to have low weights for length after comparing with the N.C.H.S reference values as required by W.H.O were then sent to the nutrition unit at the hospital as per the protocol. Double-data entry and validation were done using EpiData software (version 3.1, EpiData Association, Odense, Denmark).

### Data analysis

Means and proportions were used to describe the socio-demographic characteristics of the infants with CP+/-L and their caregiver. Nutritional characteristics were stratified using a cut off of four months which was chosen arbitrarily. Weight-for-length Z-scores were calculated using a W.H.O Z-calculator. The prevalence and severity of malnutrition was expressed as percentages with corresponding 95% Confidence Intervals. Classification of malnutrition based on severity followed the standard as recommended by WHO.

Chi-square and logistic regression was carried out for analysis of associations. All variables with p-value ≤ 0.2 were included in multivariable analysis. A significance level of 5% was considered. Unadjusted and adjusted Odds ratios with corresponding 95% CI were reported.

## Results

### Caretaker and infant characteristics

The caretakers were predominantly mothers (98%), aged less than 30 years (66%), HIV negative (79.6%), and had delivered from a Health facility (68.2%). Majority had acquired some post-delivery nutritional information from a health facility (81.5%). About half had a primary level of education (54.6%). The infants were predominantly below 4 months of age (~77%) and immunized (97.7%). The infants’ male-to-female ratio was 1.06:1. Most infants had unilateral CP+/-L (59.1%), were delivered at term (93.2%) and had a birth weight of more than 3 kg (69.4%). See Table 1.

**Table 1:** Caretaker and Infant baseline characteristics

Variable	n (%), N = 44
Caretaker Characteristics	
Median age in years [IQR]	25.5 [21.5–32.0]
Age categories of caregiver in years	
< 25	18 (40.9)
25–29	11 (25.0)
30 or +	15 (34.1)
Gender, Female	43 (97.7)
Level of education	
Tertiary	3 (6.8)
Secondary	15 (34.1)
Primary	24 (54.6)
Uneducated	2 (4.6)
HIV status	
Positive	3 (6.8)
Negative	35 (79.6)
Unknown	6 (13.6)
Acquired nutrition information post-delivery	27 (61.4)
Infant characteristics	
Median age in months (IQR)	1 [1.0–3.0]
Age categories in months	
< 4	34 (77.3)
4–10	10 (22.7)
Gender, Male	23 (52.3)
Mean birth weight in Kg (SD)	3.2 (0.8)
Birth weight category in Kg	
< 3	11 (30.6)
3 or +	25 (69.4)
Presence of other congenital anomalies	10 (22.7)
Immunized	42 (97.7)
Category of cleft	
Unilateral cleft palate and/or lip	26 (59.1)
Bilateral cleft lip and palate	18 (40.9)

### Nutritional characteristics of the infants

A total of 17 (50%) versus 4 (40%) infants below and above 4 months respectively were solely breastfeeding. Complementary feeding was reported in 17 versus 4 infants below and above 4 months respectively. Cow’s milk was the most commonly used complementary feed. About half of the mothers reported inability of the baby to suckle as the main reason for not breast feeding. See Table 2.

**Table 2:** The nutritional history of infants with cleft palate stratified by age

Variable	Age Category in Months	
	<4	4–10
	n (%)	n (%)
Most common feeding pattern		
Breast feeding	17 (50.0)	4 (40.0)
Complimentary milk	17 (50.0)	6 (60.0)
Type of complimentary milk		
Cow	13 (76.5)	6 (100.0)
Formula	4 (23.5)	0 (0.0)
Reasons for not breastfeeding		
Mother has no milk	6 (17.7)	1 (10.0)
Baby unable to suckle	19 (55.9)	5 (50.0)
Baby doesn’t take enough	2 (5.9)	1 (10.0)
No response	7(20.6)	3 (30.0)
Mean feeds per day (SD)	10 (1.5)	9.2 (1.6)
Category of feeds per day		
< 10	8 (23.5)	5 (50.0)
10 or +	26 (76.5)	5 (50.0)

### Magnitude of malnutrition

About 30 infants (68.2%; 95%CI: 53.9–82.5) were malnourished, with the highest burden among females (71.4%), infants below 4 months (73.5%) and infants with unilateral CP+/-L (77%). Generally, 57% had moderate-to-severe malnutrition (See Table 3).

**Table 3:** The Prevalence of Malnutrition

Type of Prevalence	Number	% [95% CI]
Overall Prevalence	30	68.2 [53.9–82.5]
Prevalence by severity		
Mild	5	11.4 [1.6–21.1]
Moderate	8	18.2 [6.3–30.0]
Severe	17	38.6 [23.7–53.6]
Prevalence by age (months)		
< 4	25	73.5 [57.9–89.2]
4–10	5	50.0 [12.3–87.7]
Prevalence by infants gender		
Male	15	65.2 [44.2–86.3]
Female	15	71.4 [50.4–92.3]
Prevalence by type of cleft palate		
Unilateral	20	76.9 [59.6–94.3]
Bilateral	10	55.6 [30.1–81.0]

### Associations of malnutrition

Infants whose caretakers never obtained any nutritional information post-delivery had 3.8 times higher odds of having malnutrition (OR = 3.8; [95% CI: 0.57–25.28], p = 0.17) as compared to their counterparts. Also, infants who had less than 10 feeds per day had 4.9 times higher odds of having malnutrition (OR = 4.9; [95% CI: 0.78–31.13], p = 0.09) as compared to those with above 10 feeds/day. Infants born with a low birth weight had 3.4 times higher odds of having malnutrition (p = 0.2, OR- 3.4 [95% CI: 0.52–21.71]. see Table 4.

**Table 4:** Factors influencing malnutrition among Infants with cleft palates

Variable	Normal n(%)	Malnourished n(%)	Unadjusted OR [95% CI]	Adjusted OR [95% CI]	P
Age of caregiver in years					
< 25	4 (22.2)	14 (77.8)	1		
25–29	4 (36.4)	7 (63.6)	0.5 [0.10–2.62]		
30 or +	6 (40.0)	9 (60.0)	0.4 [0.09–1.95]		
Level of education					
Tertiary	1 (33.3)	2 (66.7)	1		
Secondary	7 (46.7)	8 (53.3)	0.6 [0.04–7.74]		
Primary	6 (25.0)	18 (75.0)	1.5 [0.11–19.64]		
Uneducated	0 (0.0)	2 (100)	1		
HIV status					
Positive	2 (66.7)	1 (33.3)	1		
Negative	10 (28.6)	25 (71.4)	5 [0.41–61.52]		
Unknown	2 (33.3)	4 (66.7)	4 [0.21–75.66]		
Obtained post-delivery information					
Yes	11 (40.7)	16 (59.3)	1		
No	3 (17.7)	14 (82.4)	3.2 [0.74–13.87]	3.8[0.57–25.28]	0.17
Most common feeding pattern					
Breast milk-based	5 (23.8)	16 (76.2)	1		
Complimentary milk	9 (39.1)	14 (60.9)	0.5 [0.13–1.80]		
Type of complimentary milk					
Cow	11 (35.5)	20 (64.5)	1		
Formula	2 (33.3)	4 (66.7)	1.1 [0.17–7.00]		
Reasons for not breastfeeding					
Mother has no milk	2 (28.6)	5 (71.4)	1		
Baby unable to suckle	7 (29.2)	17 (70.8)	1.0[0.15–6.25]		
Baby doesn’t take enough	1 (33.3)	2 (66.7)	0.8 [0.04–14.64]		
Category of feeds per day					
< 10	2 (15.4)	11 (84.6)	3.5 [0.65–18.47]	4.9[0.78–31.13]	0.09
10 or +	12 (38.7)	19 (61.3)	1		
Age of baby in months					
< 4	9 (26.5)	25 (73.5)	1		
4-10	5 (50.0)	5 (50.0)	0.4 [0.08–1.54]		
Gender of baby					
Male	8 (34.8)	15 (65.2)	1		
Female	6 (28.6)	15 (71.4)	1.3 [0.37–4.78]		
Birth weight category in Kg					
< 3	2 (18.2)	9 (81.8)	3.5 [0.63–19.82]	3.4 [0.52–21.71]	0.2
3 or +	11 (44.0)	14 (56.0)	1		
Other congenital anomalies					
Yes	2 (20.0)	8 (80.0)	2.2 [0.40-11.96]		
No	12 (35.3)	22 (64.7)	1		
Category of cleft					
Unilateral cleft palate and/or lip	6 (23.1)	20 (76.9)	1		
Bilateral cleft lip and palate	8 (44.4)	10 (25.6)	0.4 [0.10-1.38]		

## Discussion

The prevalence of malnutrition among infants with CP+/-L in this study was found to be 68%. Female infants less than four months were the most affected. 50% of the infants were solely breast-fed despite having a CP+/-L, and those that were on complementary feeding were majorly on a cows’ milk diet.

The prevalence recorded in the study was higher than other studies done in other settings. The discrepancies could have been due to the differences in geographical location, methodology of assessing malnutrition and study tools.

Previous studies that documented lower prevalences were done in other geographical regions like the UK [5], Brazil [4] and India [10] which are generally at a better socioeconomic status.

In some studies, the standard deviation (Z score) method proposed by the W.H.O. was not used to define malnutrition. In the study done in Brazil [4] the preferred method of assessment was the 10^th^ percentile cut offs for documenting malnutrition whereas our study, like the study by Pandya et al used standard deviations to assess malnutrition. These differences in the methods of assessment could have led to the differences in the statistics.

Some of the studies such as the one conducted by Mehkarkar and Chaudhari [10] defined their study population differently. They excluded infants with other congenital deformities whereas we defined a participant as any infant who had a CP+/-L with or without any other congenital malformation. This could have contributed to the higher prevalence seen in our study.

The high malnutrition seen in the infants less than 4 months was probably due to the infants’ inability to breastfeed adequately. This was also noted in the study done by Cubit et al [3]. Exclusive Breast feeding is recommended for all infants less than 6 months of age by W.H.O. However, the difficulties associated with breast feeding among infants with CP+/-L are well documented [6]. Therefore, assisted feeding for infants with CP+/-L is encouraged as it has been found to be more reliable [11]. The choices available for complementary feeding vary from cow’s milk to infant formula. However, breast milk is still regarded as the milk of choice for its numerous advantages including protection against otitis media in cleft palate infants [12]. Numerous methods have been described for assisted feeding for these infants including compressible bottles, modified nipples, cups and prostheses or appliances. However, the evidence to support one particular method over the others has been found to be weak [6]. As the methods of assisted feeding were not assessed in this study, another study may therefore be required to assess the most applicable feeding method to assist infants born with CP+/-L in our setting in order to reduce the rate of malnutrition.

The prevalence of malnutrition among females was higher than that of males and the overall prevalence. The cause of this imbalance needs further investigation as very few studies evaluating the problem have focused on gender differences. Literature from elsewhere seem to point to a gender imbalance as regards to children’s nutrition and access to health care. A study done in Pakistan showed that male children were favored more in the allocation of health care compared to the females [13]. Could the same cultural anomaly have led to more access to health care for the male infant compared to the female and therefore contributed to more malnourished females? Also, most of the mothers in our study were educated up to primary school level. The fact that they did not get further education could have limited their access to better education against some of these cultural anomalies.

Malnutrition was also found to be higher among the unilateral CP+/-L infants compared to their bilateral counterparts. Could the severe facial disfigurement and apparent difficulty in breast feeding in bilateral CP+/-L have forced the mothers to seek medical attention earlier than their unilateral counterparts? This could have led to the differences observed.

Regarding the associated factors, the lack of provision of nutritional information was associated with malnutrition among our infants with CP+/-L. Provision of nutritional information has been shown to be associated with improved outcomes. In the study performed in the United Kingdom [5], they noted a decline in malnutrition after instituting a feeding support nurse whose role was to provide nutritional information and follow up among other feeding interventions.

Our study noted that children who had fewer than 10 feeds a day were more likely to be malnourished. This could have been due to failure of the infant to breastfeed. The most common reason for not breast feeding was failure of the infants to suckle (Table 3) which has been documented elsewhere [14] as a common reason for not breastfeeding infants with a cleft palate. Even though it was not investigated, the fear of the task of feeding their cleft palate infants has been documented as a contributor for malnutrition [6].

The Study showed that the risk of malnutrition was higher for CP+/-L infants born with a low birth weight. This finding is similar to studies done in Oman [15] and Malawi [16]. There is need therefore for the integration of other health services to ensure mothers deliver healthy infants as maternal environmental factors such as smoking, indoor air pollution, infections such as malaria as well as underlying social factors such as poverty are important contributors to low birth weight [17].

The combination of lack of provision of nutritional information following the delivery of a CP+/-L infant, number of feeds less than 10 received per day by the CP+/-L infant and low birth weight was found to be the greatest predictor of malnutrition among CP+/-L infants.

This is the first study in our setting to explore the prevalence and severity of malnutrition with its associated factors among infants with CP+/-L.

## Conclusions

This work described malnutrition among infants with CP+/-L at a specialized plastic surgery hospital in Uganda.

Like other studies previously reported, there was a high prevalence of malnutrition in this population. The low study participants obtained for the study was a major study limitation. However, the Low recruitment of study subjects concurred with studies done in a similar setting that showed few infants with CP+/-L were being seen at the time of their operation probably because they were dying earlier [8]. Malnutrition was hypothesized to have contributed to their deaths. Even though the low numbers could have affected the power of the study, the high prevalence of malnutrition among CP+/-L infants showed clinical significance.

We managed to describe the severity of malnutrition according to the WHO classification for malnutrition which no other studies had done in our setting. The fact that most children had severe malnutrition further strengthened the need for aggressive nutritional management for infants with CP+/-L with the help of the motivated caretaker.

Many of the factors associated with CP+/-L were directly related to the caregiver status. There is therefore need for integration of everyday healthcare practices of the caregivers into CP+/-L surgical services to reduce the risk of malnutrition among these infants.

Finally, longitudinal and interventional studies will be needed to better determine causation in many of the factors noted and to improve outcomes so that we prevent malnutrition in infants with CP+/-L.

## Recommendations

Proper Nutritional information should be readily available for all mothers who give birth to infants with CP+/-L.

The nutritional information available should include methods of assisted feeding, feeding techniques such as positioning and follow up. Further studies may be required to assess the different modalities for assisted feeding in order to come up with the most applicable method for these caretakers to adopt in our setting.

There is need to increase public awareness of the likely hood of malnutrition among infants born with a cleft palate. This would help emphasize nutrition both for the caretakers and care providers of these infants.

Further studies are required to elucidate the role of culture in access of health care as regards the gender of the infants and perceptions of the cleft conditions.

There is need to strengthen the link between general health care and maternal health care in our setting. We need to re-emphasize for example the need for proper maternal nutrition, the avoidance of smoking in pregnancy among others.

## Abbreviations

CL: Cleft lip
CoRSU: Comprehensive Rehabilitation Services for Uganda
CP+/-L: Cleft palate with or without Cleft Lip
M.U.S.T: Mbarara University of Science and Technology
N.C.H.S: National Centre for Health Statistics
W.H.O: World Health Organization
